# Intra-articular injection of monoiodoacetate induces diverse hip osteoarthritis in rats, depending on its dose

**DOI:** 10.1186/s12891-022-05454-y

**Published:** 2022-05-25

**Authors:** Satoshi Yoh, Yuya Kawarai, Shigeo Hagiwara, Sumihisa Orita, Junichi Nakamura, Shuichi Miyamoto, Takane Suzuki, Tsutomu Akazawa, Yuki Shiko, Yohei Kawasaki, Seiji Ohtori

**Affiliations:** 1 Department of Orthopaedic Surgery, Graduate School of Medicine, Chiba University, 1-8-1 Inohana, Chuo-ku, Chiba City, Chiba 260-8677 Japan; 2 Department of Orthopaedic Surgery, Matsudo City General Hospital, 993-1 Sendabori, Matsudo City, Chiba 270-2296 Japan; 3 Department of Bioenvironmental Medicine, Graduate School of Medicine, Chiba University, 1-8-1 Inohana, Chuo-ku, Chiba City, Chiba 260-8677 Japan; 4 Department of Orthopaedic Surgery, St. Marianna University School of Medicine, 2-16-1 Sugao, Miyamae-ku, Kawasaki, Kanagawa 216-8511 Japan; 5 Biostastics Section, Clinical Research Center Chiba University Hospital, 1-8-1 Inohana, Chuo-ku, Chiba City, Chiba 260-8677 Japan

**Keywords:** Monoiodoacetate (MIA), Hip OA, Hip pain, Animal model

## Abstract

**Background:**

Monoiodoacetate (MIA)-induced arthritis models are used widely in osteoarthritis (OA) research to develop effective conservative treatments for hip OA, as an alternative to joint replacement surgery. In joint OA models, such as the MIA-induced knee OA model, various doses of MIA are utilized, depending on the purpose of the research. So far, only 2 mg of MIA has been used for MIA-induced hip OA research. We hypothesized that the amount of MIA should be adjusted according to the osteoarthritis model under investigation. We performed radiographic and histological evaluations in rats for hip OA models induced by different doses of MIA.

**Methods:**

One hundred and eighty right hips of six-week-old, male Sprague–Dawley rats (*n* = 30 rats per group) were treated with either a single intra-articular injection of various doses of MIA (0.25, 0.5, 1.0, 2.0, and 4.0 mg) dissolved in 25 μl of sterile saline (MIA group), or with 25 μl of sterile saline alone (Sham group). Radiographic and histological evaluations of the hip joint were performed at one, two, four, eight, and 12 weeks after administration (*n* = 6 rats per group per time point).

**Results:**

OA changes progressed from 1 week after administration in the 1.0-mg, 2.0-mg, and 4.0-mg MIA groups. The degree of OA changes increased as the dose of MIA increased. The 0.25-mg and 0.5-mg MIA groups presented fewer OA changes than the 2.0-mg and 4.0-mg MIA groups during the entire study period (up to 12 weeks). The administration of 0.25 mg and 0.5 mg of MIA-induced both radiographic and histological OA changes in a time-dependent manner, whereas more than 2 mg of MIA provoked end-stage OA at 8 weeks after injection. Absolute, dose-dependent histopathological OA changes were observed 4 weeks after MIA administration.

**Conclusions:**

Intra-articular MIA injection to the hip joints of rats induced diverse OA changes dose-dependently. Research for developing novel conservative treatments for hip OA and intractable pain should consider the pathological condition when determining the dose of MIA to be employed.

## Background

Osteoarthritis (OA) is a major musculoskeletal disorder that affects at least half of the elderly population [1]. Hip and knee OA is rank globally as a significant cause of disorders [2]. Wage losses due to OA amount to US$65 billion; direct medical costs exceed US$100 billion [3]. It is estimated that more than 24 million people worldwide suffer from symptomatic and activity-restricting hip OA [4, 5]. Patients with hip OA who visit clinics usually complain of hip pain rather than dysfunction (such as limited range of motion or contracture). However, the pain mechanism in osteoarthritis is not yet completely understood. Previous reports have attributed pain to avascular necrosis of the femoral head and hip. OA appears not only in the inguinal region but also in various other locations, such as the anterior thigh and buttocks, making diagnosis difficult [6, 7]. It is important to realize that pain in hip OA is not only derived from bones and cartilages but also from soft tissues, such as the acetabular labrum [8]. In clinical practice, most patients whose hip pain is refractory to symptomatic treatments (such as nonsteroidal anti-inflammatory drugs and rehabilitation) usually require total hip arthroplasty (THA). Increasing medical costs due to increasing THAs is also a health economics problem. Since most patients with OA are elderly and often have multiple comorbidities [3], a certain percentage of patients cannot undergo surgery. Therefore, from the viewpoint of both health economics and patient care, we need to develop disease-specific treatments for hip OA.

A few hip OA models have been proposed for the development of new therapeutic agents for hip pain [8, 9]. The MIA intra-articular injection model is commonly utilized for research on OA and OA related pain. Intra-articular injection of 2 mg of MIA into the hip joint induced end-stage hip OA and increased the expression of calcitonin gene-related peptide, an inflammatory neuropeptide in dorsal-root ganglia. It activated transcription factor-3, a selective marker of cell damage following nerve injury [10]. The expression of microglia in the spinal cord also increased in this OA model. The systematic administration of serotonin and norepinephrine reuptake inhibitor attenuated hip pain via descending pain modulatory system, which suggested that end-stage OA is associated with both inflammatory and neuropathic pain [11]. On the other hand, intra-articular administration of 2 mg of MIA provoked relatively strong joint instability, which was different from the gradual OA changes seen in clinical practice. In the MIA-induced knee OA model, it has been reported that the degree of OA varies depending on dose and time after MIA administration [12]. There are no reports describing how osteoarthritic changes of the hip joint proceed depending on the dose of MIA and time course. We hypothesized that the amount of MIA should be adjusted according to what kind of osteoarthritis model is required. We created MIA-induced hip OA models induced by different MIA doses, then evaluated radiographic and histological findings at multiple time points.

## Methods

All research protocols in this study were reviewed and approved by the Chiba University Institutional Animal Care and Use Committee and were performed in compliance with the ARRIVE guidelines.

### Intra-articular injection of MIA

One hundred eighty male Sprague–Dawley rats (CLEA, Tokyo, Japan), weighing 250–300 g, were housed in a semi-barrier system with a controlled environment (12 h/12 h light/dark cycle; temperature, 21 °C–23 °C; humidity: 45–65%). Animals were given free access to food and water on arrival. All animals were fed a diet of standard rodent chow (CRF-1; Oriental Yeast, Tokyo, Japan). On the basis of methods previously published [13], all animals were anesthetized using an intraperitoneal injection of 0.3 mg/kg of medetomidine, 4.0 mg/kg of midazolam, and 5.0 mg/kg of butorphanol. All animals were treated aseptically throughout the experiments. Using a 27-gage needle, various concentrations of MIA (Sigma-Aldrich, St. Louis, MO, USA), or sterile saline, were injected into the right hip joints using a posterior approach [9, 14].

Five groups of rats (*n* = 30 per group) were treated with a single intra-articular injections into the hip joint of various doses of MIA (0.25, 0.5, 1.0, 2.0, and 4.0 mg) dissolved in 25 μl of sterile saline (MIA groups). Thirty rats were injected with 25 μl of sterile saline only (Sham group). The injected agent was confirmed to be restricted to the joint cavity. The animals were examined radiologically and histologically at one, two, four, eight, and 12 weeks after administration (*n* = 6 rats per group per time point). The rats were euthanized using 3 g of potassium chloride dissolved in 50 ml of sterile saline, administered intravenously (2–4 ml) to anesthetized rats.

### X-ray imaging and tissue preparation of the hip joint

Tissue preparations and X-ray imaging of the hip joint were performed as previously described by Miyamoto et al. [9]. After intraperitoneal anesthesia, the rats were laid in the supine position with 0° of hip flexion, abduction, and internal and external rotation. Anteroposterior bilateral X-rays of the hips were taken using the In-Vivo Xtreme imaging system (Bruker, Billerica, MA, USA). Lateral images were taken at 45° of flexion and abduction and at 0° of internal and external rotation.

Radiographic assessments were classified using the Kellgren and Lawrence (KL) system [15] as grade 0 (none: no radiographic features of OA), grade 1 (doubtful: doubtful joint space narrowing [JSN] and possible osteophyte), grade 2 (minimal: the presence of definite osteophytes and possible JSN), grade 3 (moderate: multiple osteophytes, definite JSN, sclerosis, possible bony deformity), and grade 4 (severe: large osteophytes, marked JSN, severe sclerosis and definite bony deformity).

For histological evaluation, the rats were intraperitoneally anesthetized, as described above, and perfused transcardinally with 0.9% saline, followed by 500 ml of 4% paraformaldehyde in phosphate buffer fixative (0.1 M, pH 7.4). The soft tissues around the right hip joint (cartilage, synovium, and capsule) were resected. The resected limbs were cut at the mid-femur level and at the center of the femoral head, then immersed in 10% neutral buffered formalin for 3 days. The specimens were continuously demineralized in reagent K-CX (Falma, Tokyo, Japan) for 30 h and in 5% sodium sulfate for 16 h, then paraffin-embedded for coronal sectioning. The samples were serially sectioned in steps of 8-μm and stained using hematoxylin and eosin, Safranin O, and Toluidine Blue. Osteoarthritic changes were evaluated using the Osteoarthritis Research Society International (OARSI) histopathology score [16]. For each joint, we scored ten slices centered on the maximum diameter of the femoral head. Each sample was assessed by the depth (grading) and width (staging) of the osteoarthritic changes. The final score was obtained by multiplying the depth and width.

### Statistical analysis

Because grades and OARSI scores were measured in different rats at each time point, we performed two-way analysis of variance (ANOVA). We compared the data between groups at each time point and between time points within each group. Pairwise differences of least-squared means were calculated. The Tukey–Kramer method was used to adjust *p*-values. A *p*-value of < 0.05 was considered statistically significant. Statistical analyses were performed using SAS statistical software (version 9.4; SAS Institute, Cary, NC, USA).

## Results

### X-ray findings

**Fig. 1:**
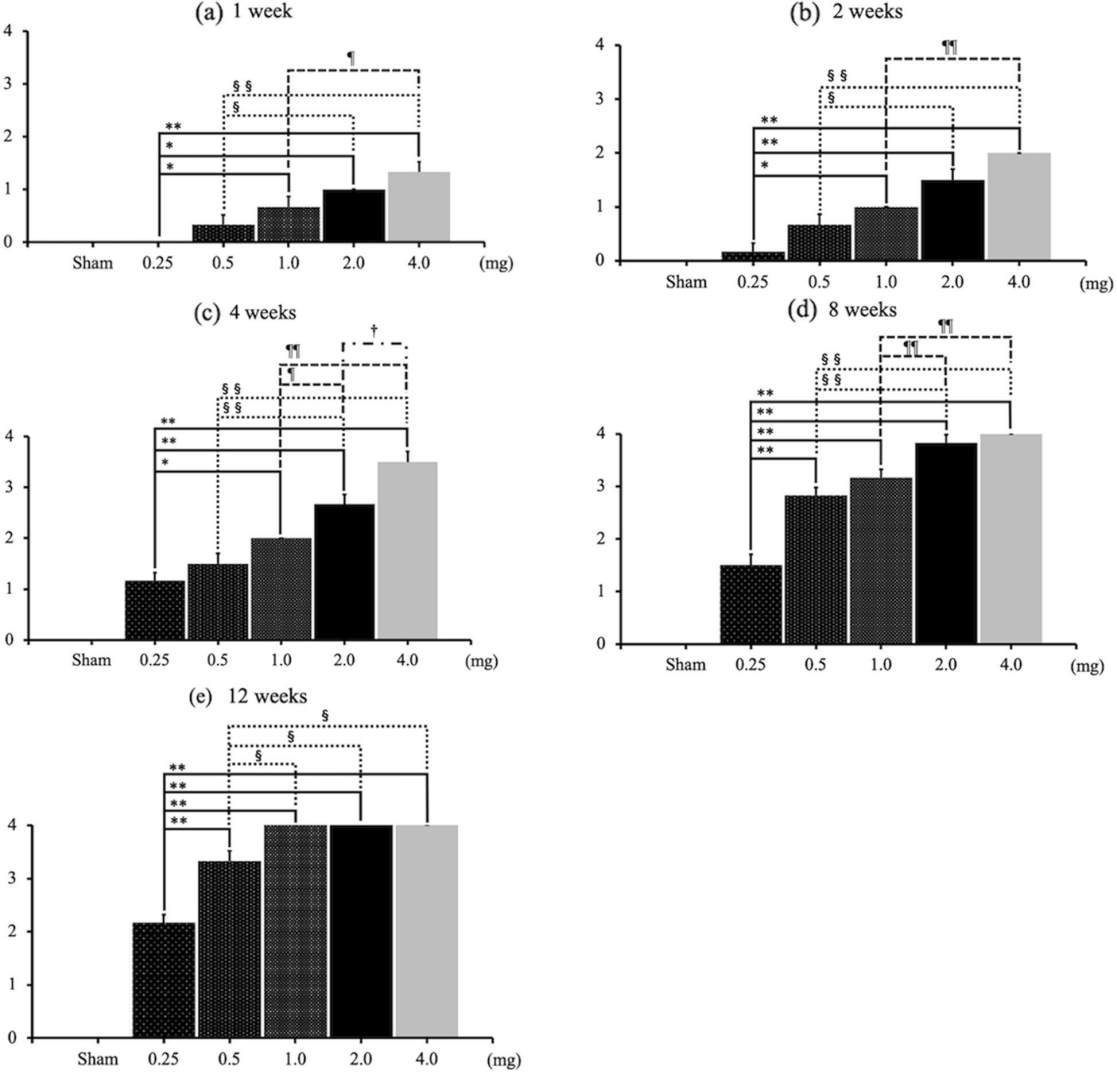
KG grades at each time point. **a**–**e** The KL classification grades at each time point, based on the dose of MIA. At each time point, the KL grades in the 2.0-mg and 4.0-mg MIA groups were significantly higher than those in the 0.25-mg and 0.5-mg MIA groups. At 8 weeks after MIA injection, the KL grades approached 4 in the groups receiving 2 mg MIA or more. At 12 weeks, the KL grades reached 4 in the groups receiving more than 1 mg of MIA. Data are presented as mean ± SEM, *n* = 6 rats per group. Data were analyzed using two-way ANOVA, followed by the Tukey–Kramer method. *, *p* < 0.05; **, *p* < 0.01 (vs. 0.25-mg MIA group). §, *p* < 0.05; §§, *p* < 0.01 (vs. 0.5-mg MIA group). ¶, *p* < 0.05; ¶¶, *p* < 0.01 (vs. 1.0-mg MIA group). †, *p* < 0.05; ††, *p* < 0.01 (vs. 2.0-mg MIA group). **Abbreviations:** KL, Kellgren and Lawrence system; MIA, monoiodoacetate; SEM, standard error of the mean

**Fig. 2:**
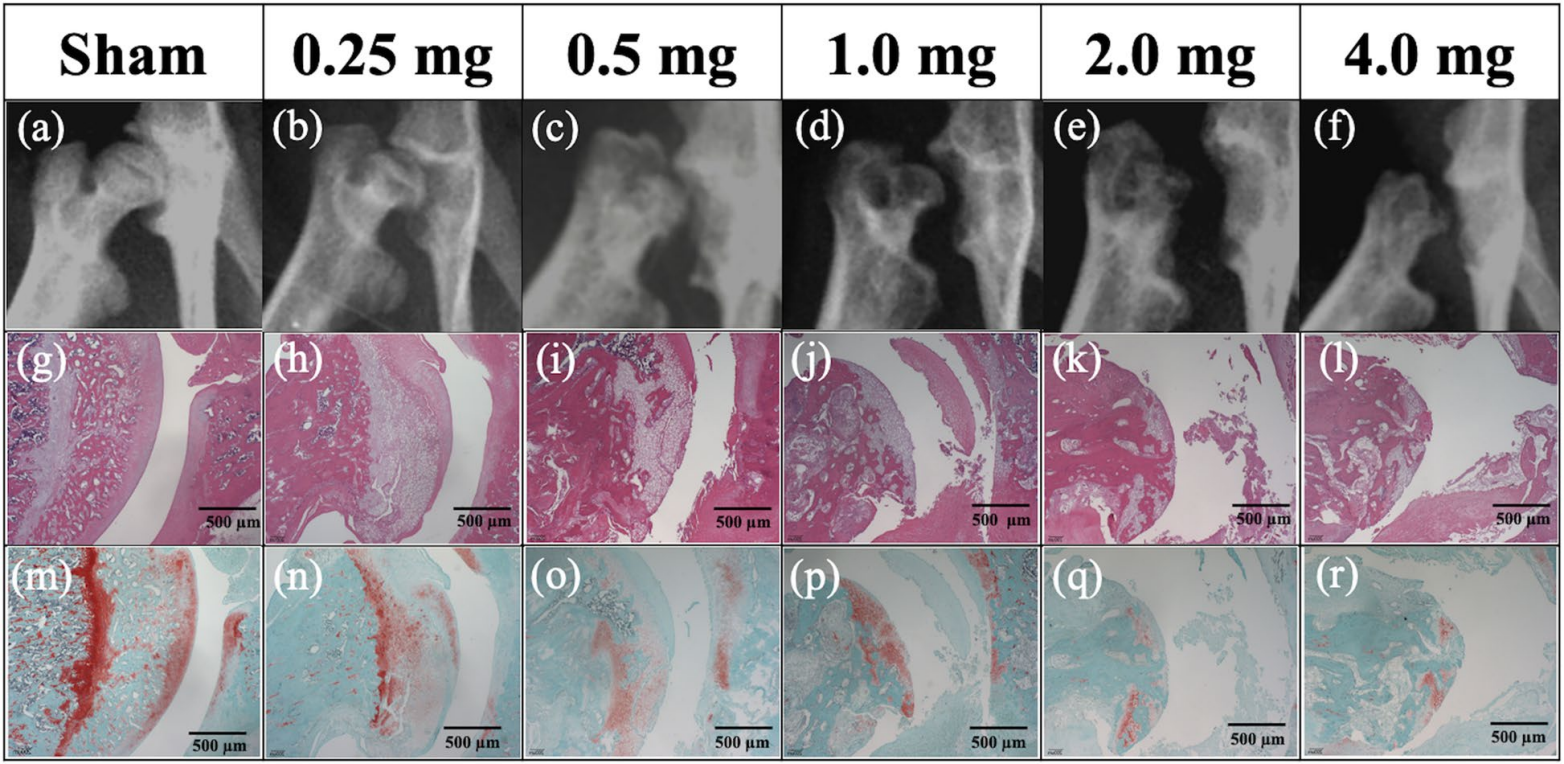
Radiography and histopathology of the hip. Radiography and histopathology of the hip in the Sham and MIA groups (0.25-mg, 0.5-mg, 1.0-mg, 2.0-mg, and 4.0-mg) 12 weeks after MIA administration. **a**–**f** Anteroposterior radiographs of the rat hip. **g**–**l** Hematoxylin and eosin-stained tissues. **m**–**r** Safranin O-stained tissues. As the MIA dose increased, OA changes progressed both radiologically and histologically. At concentrations of 0.5 mg MIA and higher, end-stage joint destruction was observed at 12 weeks. **Abbreviations:** MIA, monoiodoacetate; OA, osteoarthritis

**Fig. 3:**
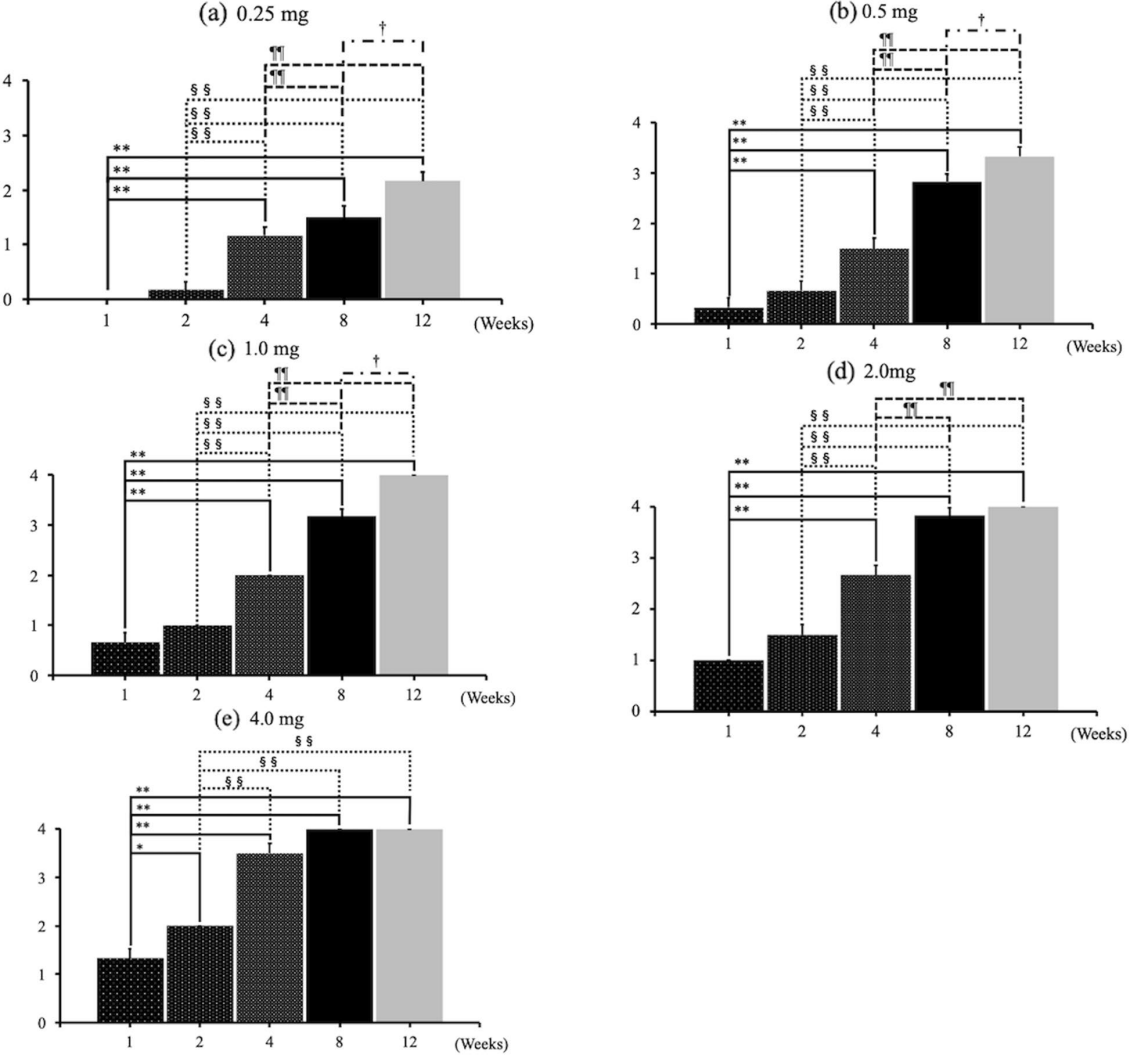
KL grades, based on MIA dose. **a**-**c** KL grades increased in a time-dependent manner in the 0.25-mg, 0.5-mg, and 1.0-mg MIA groups. **d**–**e** However, in the 2-mg and 4-mg MIA groups, the KL grades reached a plateau 8 weeks after administration. Data are presented as mean ± SEM, *n* = 6 rats per group. Data were analyzed using two-way ANOVA, followed by the Tukey–Kramer method. *, *p* < 0.05; **, *p* < 0.01 (vs. 1 week group). §, *p* < 0.05; §§, *p* < 0.01 (vs. 2-week group); ¶¶, *p* < 0.01 (vs. four-week group). †, *p* < 0.05 (vs. eight-week group). **Abbreviations:** KL, Kellgren and Lawrence system; MIA, monoiodoacetate; SEM, standard error of the mean

OA changes progressed from 1 week after administration in the 1.0-mg, 2.0-mg and 4.0-mg MIA groups. The degree of OA changes increased as the dose of MIA increased (Fig. 1a). At each time point, the higher the dose of MIA, the stronger the degree of change in the joint (Fig. 1a–e). At 8 weeks after MIA injection, the KL classification grades were nearly equal to 4 in the groups receiving 2 mg or more of MIA (Fig. 1d). At 12 weeks, the KL classification grades reached 4 in the groups receiving more than 1 mg of MIA (Fig. 1e). At the end of the 12th week, progressive to end-stage hip OA changes were observed, except in the Sham and 0.25-mg MIA groups (Fig. 1e, Fig. 2a–r). At 4 weeks after administration, completely dose-dependent OA changes were found (Fig. 1c). For hips injected with the same dose of MIA, KL classification grades increased in a time-dependent manner in all groups (Fig. 3a–e). At 12-weeks after administration, the 0.25-mg MIA group displayed slight OA (Fig. 3a), the 0.5-mg and 1.0-mg MIA groups presented mild to severe OA (Fig. 3b and c). In the 2-mg and 4-mg MIA groups, the KL classification grades reached a plateau at 8 weeks after administration (Fig. 3d and e).

### Histopathological findings

**Fig. 4:**
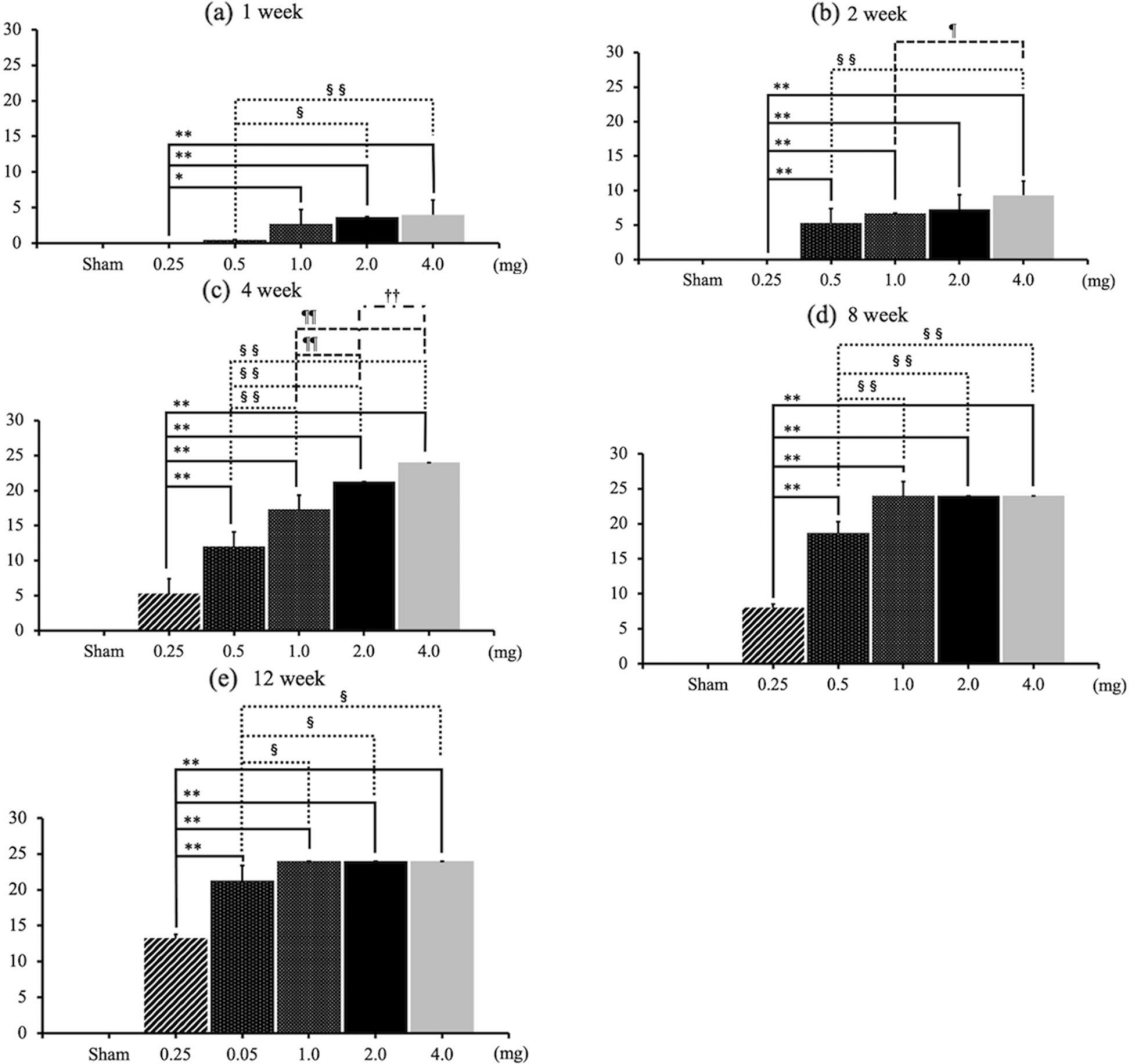
OARSI scores at each time point. **a**–**b** OARSI scores in the 4.0-mg MIA group at one and 2 weeks after MIA administration were significantly higher than those in 0.25-mg and 0.5-mg MIA groups. **c** In the fourth week, OARSI scores increased in a completely dose-dependent manner. **d**–**e** After the eighth week, there were no significant differences between the 1.0-mg, 2.0-mg, and 4.0-mg MIA groups. Data are presented as mean ± SEM, *n* = 6 rats per group. Data were analyzed using two-way ANOVA, followed by the Tukey–Kramer method. *, *p* < 0.05; **, *p* < 0.01 (vs. 0.25-mg MIA group). §, *p* < 0.05; §§, *p* < 0.01 (vs. 0.5-mg MIA group). ¶, *p* < 0.05; ¶¶, *p* < 0.01 (vs. 1.0-mg MIA group). ††, *p* < 0.01 (vs. 2.0-mg MIA group). **Abbreviations:** OARSI, Osteoarthritis Research Society International; MIA, monoiodoacetate; SEM, standard error of the mean

**Fig. 5:**
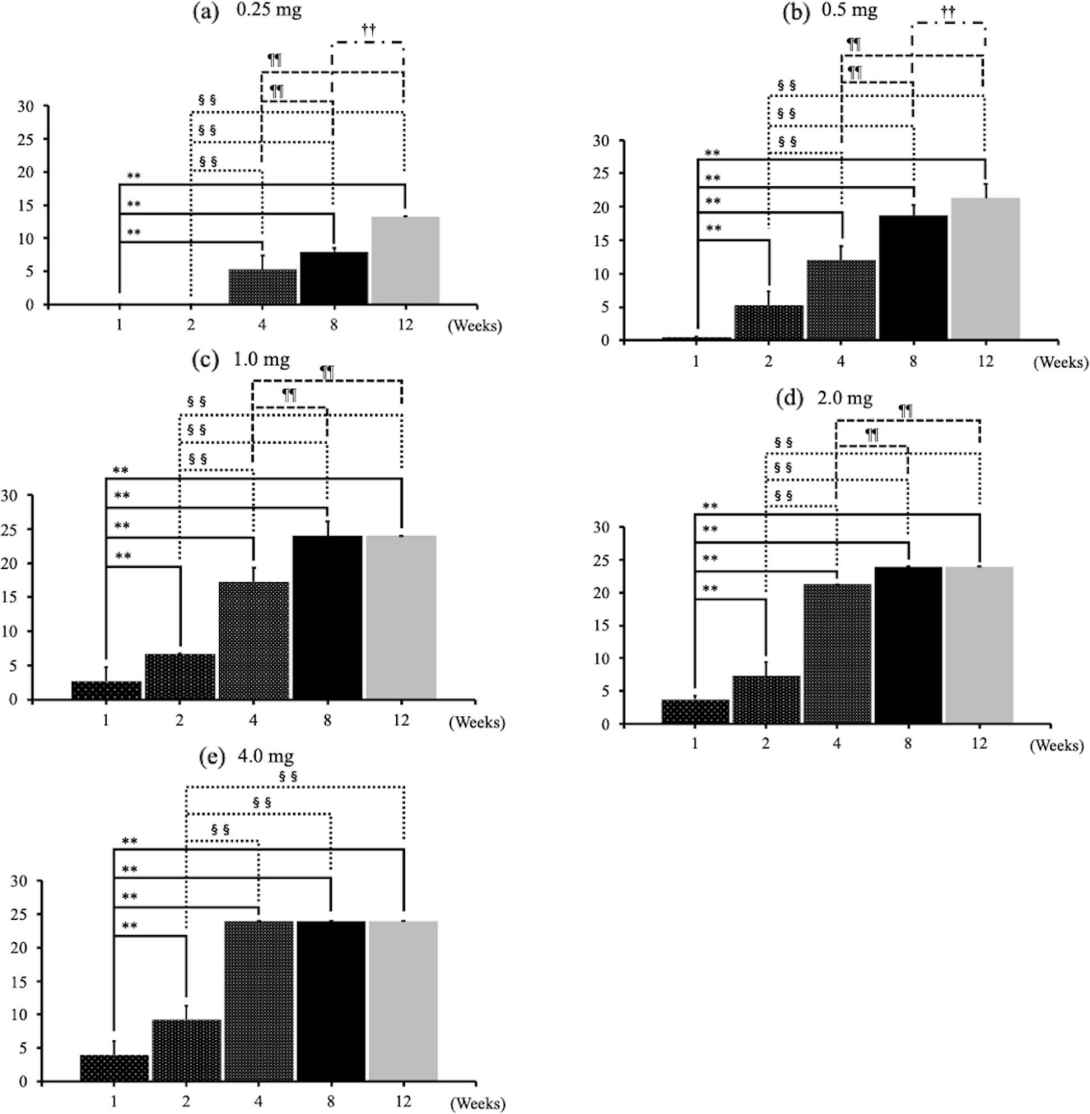
OARSI scores, based on MIA dose. **a**–**b** With 0.25 mg and 0.5 mg of MIA, OARSI scores increased in a time-dependent manner, except for the score at 1–2 weeks in the 0.25-mg MIA group. **c**–**d** With 1.0 mg and 2.0 mg of MIA administration, time-dependent increases in OARSI scores were observed until 4 weeks after MIA injection. e With 4.0 mg of MIA administration, OARSI scores reached the maximum after 4 weeks. There were no significant differences among the four-week, eight-week, and 12-week groups. Data are presented as mean ± SEM, *n* = 6 rats per group. Data were analyzed using two-way ANOVA, followed by the Tukey–Kramer method. **, *p* < 0.01 (vs. 1 week group). §§, *p* < 0.01 (vs. two-week group). ¶¶, *p* < 0.01 (vs. four-week group). ††, *p* < 0.01 (vs. eight-week group). **Abbreviations:** OARSI, Osteoarthritis Research Society International; MIA, monoiodoacetate; SEM, standard error of the mean

At 1 week after MIA administration, the OARSI scores of the 2.0-mg and 4.0-mg MIA groups were significantly higher than those of the 0.25-mg and 0-5 mg MIA groups (Fig. 4a). They were also higher than those of the 0.25-mg MIA group at 2 weeks after administration (Fig. 4b). At 4 weeks after administration, completely dose-dependent OA changes were observed (Fig. 4c). At eight and 12 weeks after administration, more than 1.0 mg of MIA provoked complete OA changes (Fig. 4d and e, Fig. 2g-r). In rats injected with the same doses of MIA, increases in OARSI scores occurred in a time-dependent manner (Fig. 5a and b). The OARSI scores reached a maximum at 8 weeks after MIA injection in the 1.0-mg and 2.0-mg MIA groups, and at 4 weeks after injection in the 4.0-mg MIA group (Fig. 5c–e).

## Discussion

Ours is the first study to investigate radiographic and histological changes after intra-articular injection of various concentrations of MIA to hip joints. Overall, radiographic findings were consistent with histologic findings. OA changes progressed from 1 week after administration in the 1.0-mg, 2.0-mg, and 4.0-mg MIA groups. The degree of OA changes increased as the dose of MIA increased. The 0.25-mg and 0.5-mg MIA groups presented fewer OA changes than the 2.0-mg and 4.0-mg MIA groups over the entire study period of 12 weeks. Interestingly, explicit, dose-dependent, histopathological OA changes were observed 4 weeks after MIA administration. OA progression was noted over time in the 0.25-mg and 0.5-mg MIA groups. More than 2 mg of MIA administration provoked end-stage OA at 8 weeks after MIA injection.

Our radiographic findings were consistent with our histological findings. In a previous study of MIA-induced hip OA, rats injected with 2.0 mg of MIA were radiographically shown to have severe OA 28 days after induction. The histological OARSI scores were 22.7. Thus, the radiographic and histological outcomes were similar [11]. Our results are consistent with these. Another study of MIA-induced hip OA reported that injection of 2 mg of MIA to the hip joints provoked time-dependent OA changes, both radiographic and histologic [9]. In that study, the KL classification grades were 2, 3, and 4, respectively, on days 14, 28, and 56 after MIA administration. The Mankin scores (a histopathological classification of OA severity, ranging from 0 to 14) were 5.0, 8.5, and 12.7, respectively, on days 14, 28, and 56 after MIA injection. Thus, radiographic outcomes were consistent with histological outcomes. This is also consistent with our findings.

OA changes progressed after 1 week of treatment in the 1.0-mg, 2.0-mg, and 4.0-mg MIA groups. The degree of OA changes increased as the MIA dose increased. To the best of our knowledge, no previous report has clarified dose-dependent OA change after MIA injection to the hip joints. In MIA-induced knee OA models, it has been reported that histological OA changes are induced in a dose-dependent manner [12]. MIA causes a local acute inflammation, followed by the degeneration of cartilage [17, 18]. Kawarai et al. reported that the concentrations of tumor necrosis factor-α (from the synovium of rats injected with 2.0 mg of MIA to the hip joints) significantly increased from Day 7 after induction [14]. A recent study in a MIA-induced knee OA model, using 1.0 mg, 2.0 mg, and 3.0 mg of MIA, explored the levels of chemokines (a family of cytokines that play a crucial role in inflammation and immunity) [19]. The protein levels of chemokine (C-C motif) ligand 2 (CCL2) and chemokine (C-X-C motif) ligand 1 (CXCL1) were increased in synovial fluid from Day 2 after MIA injection into the knee joint. This was more apparent in the 3.0-mg MIA group, compared with the 1.0-mg MIA group [20]. OA progression is associated with inflammation [21]. Thus, it is reasonable that OA changes were observed after 1 week of treatment in the 1.0-mg, 2.0-mg, and 4.0-mg MIA groups, and that the degree of OA changes increased as the MIA dose increased.

From the first to the 12th week after administration, the 0.25-mg and 0.5-mg MIA groups displayed fewer OA changes, both radiographic and pathologic, than the 2.0-mg and 4.0-mg MIA groups. OA progression over time was noted in the 0.25-mg and 0.5-mg MIA groups. More than 2 mg of MIA administration provoked end-stage OA at 8 weeks after injection. No previous reports have used a hip OA model to evaluate changes over time in groups treated with different MIA concentrations. In the MIA-induced knee OA model, Udo et al. clarified dose- and time-dependence in MIA-induced OA in rats. MIA concentrations of 0.1 mg, 0.2 mg, and 0.5 mg induced macroscopic cartilage loss in a concentration-dependent manner, which progressed over time from two to 8 weeks after administration [12]. Janusz et al. injected various doses (0.1, 0.25, 0.5, and 1.0 mg) of MIA into the knee joint and performed the histological assessment 3 weeks after induction. Mild cartilage damage was observed at the low dose of 0.1 mg of MIA, whereas severe damage was observed at the high dose of 1 mg. Cartilage damage had proceeded for 3 weeks at the dose of 0.25 mg of MIA [22]. Another study revealed that the administration of 1.0 mg of MIA into the knee joint provoked time-dependent OA. Focal fragmentation and collapse of bony trabeculae with fibrosis and increased osteoclastic activity were observed by 28 days [23]. Guingamp et al. performed functional and histological evaluations at 2 weeks and 4 weeks after administering MIA at high doses (0.3 mg, 3.0 mg) and low doses (0.01 mg, 0.03 mg, and 0.1 mg) [24]. They reported that locomotor activity decreased and tissue degeneration increased in high-dose groups, compared with low-dose groups. Fonsi et al. described that the low dose of less than 0.3 mg of MIA injection into the rat knee joint did not attain an appreciable impairment level within 7 days after injection, whereas more than 2 mg of MIA administration led to severe irreversible damage within 14 days [25]. Similarly, In the current study on the hip joint, the 0.25-MIA group did not display any histological OA change. In a rat knee joint model, Ferreira et al. showed that nerve injury markers ATF-3 and NPY (in nerve roots) increased in a dose-dependent manner after administration of MIA at different concentrations (0.3 mg–2 mg) [26]. Kanno et al. compared the literature on histopathological evaluation at 8 weeks after MIA administration to the hip joint. They reported that, in rats with hip OA induced by 0.5 mg of MIA, OARSI scores were approximately 10 at 4 weeks after induction [27]. By contrast, another study reported that OARSI scores were almost at maximum 4 weeks after administration of 2 mg of MIA into the hip joint [11]. These results validate our findings, i.e., more than 2 mg of MIA provoked end-stage OA at 8 weeks after injection. An interesting result of our study is that completely dose-dependent histopathological OA changes were observed 4 weeks after MIA administration. This provides scientists with evidence regarding when to evaluate results for comparison between different concentration of MIA.

### Clinical relevance

This is the first research to examine histologic and radiographic changes in hip OA over time after administration of different concentrations of MIA. A previous study investigated OA changes in hip joints over time after administration of a single concentration of 2 mg MIA [9]. Our study had a longer observation period (12 weeks), which allowed us to explore the fundamental pathology of OA in clinical practice, characterized by the disruption of chondrocyte homeostasis (cracking and erosion), synovitis, exposure of subchondral bone, and osteophyte formation. By applying the results of this study, it is possible to reproduce various types of OA. For example, a low concentration of MIA (< 0.5 mg) should be used to reproduce mild OA; a high concentration of MIA (> 2.0 mg) should be used to replicate vigorous deformities, such as pyogenic arthritis, rapidly destructive coxarthrosis, and destruction of the femoral head via rheumatoid arthritis. Moreover, our findings could be useful for elucidating the complex mechanism of pain in OA patients. Research has increasingly emphasized the role of central sensitization in OA, in addition to peripheral sensitization [28–31]. We believe that such research could contribute to elucidation of the mechanism of intractable pain, which significantly impairs the quality of life of OA patients. This may lead eventually to the development of novel treatments for refractory OA pain.

## Conclusion

Intra-articular injection of MIA to the hip joints of rats induced diverse OA changes dose-dependently. For research to develop novel conservative treatments for hip OA and intractable hip pain, it is necessary to employ a suitable dose of MIA, according to the pathological condition under investigation.

## Data Availability

The datasets used and/or analyzed during the current study are available from the corresponding author on reasonable request.
